# Serum Small Proline-Rich Protein 2A (SPRR2A) Is a Noninvasive Biomarker in Gastric Cancer

**DOI:** 10.1155/2020/8493796

**Published:** 2020-08-28

**Authors:** Xiaoming Xu, Shumei Wei, Yueming Chen, Daojun Yu, Xianjun Wang, Xueyan Dong

**Affiliations:** ^1^Department of Pathology, The Second Affiliated Hospital of Zhejiang University School of Medicine, No. 1511 Jianghong Road, Hangzhou, 310051 Zhejiang Province, China; ^2^Department of Clinical Laboratory, Affiliated Hangzhou First People's Hospital of Zhejiang University School of Medicine, No. 261 Huansha Road, Hangzhou, 310006 Zhejiang Province, China

## Abstract

**Objective:**

Since early diagnosis is very important for treating gastric cancer (GC), we aimed to detect serum small proline-rich protein2A (SPRR2A) to verify its diagnostic value for GC patients.

**Methods:**

Serum samples were collected from 200 patients with GC, 100 patients with gastritis, 40 patients with rectal cancer (RC), 50 patients with colon cancer (CC), and 100 healthy controls. An enzyme-linked immunosorbent assay (ELISA) detection kit was applied to measure serum SPRR2A concentration. The correlations between serum SPRR2A and carcinoembryonic antigen (CEA), clinical pathological parameters of GC, and receiver operating characteristic (ROC) curve were also analyzed.

**Results:**

The median serum SPRR2A concentration in GC patients was significantly higher than those in healthy controls and gastritis or colorectal cancer patients (*P* < 0.001). Serum SPRR2A concentration at a cut-off value of 80.7 pg/ml yielded an AUC of 0.851, with 75.7% sensitivity and 74.5% specificity for discriminating GC patients from healthy people. The AUC for the serum SPRR2A concentration combined with the CEA concentration was 0.876, with 79.7% sensitivity and 78.7% specificity. Similarly, serum SPRR2A discriminated GC patients from gastritis patients with an AUC of 0.820, with 90.5% sensitivity and 61.7% specificity. The AUC for the serum SPRR2A concentration combined with the CEA concentration was 0.848, with 87.8% sensitivity and 68.1% specificity. The serum SPRR2A levels in GC patients were associated with lymph node metastasis and the tumor-node-metastasis (TNM) stage (*P* < 0.05). There was an obvious difference in serum SPRR2A expression between GC patients before and after surgery (*P* < 0.0001).

**Conclusion:**

These results suggest that serum SPRR2A can be used as an effective marker for GC.

## 1. Introduction

Gastric cancer (GC), an epithelial malignancy, is the third leading cause of cancer-related deaths globally, accounting for 7% of cancer cases and 9% of cancer-related deaths [1]. The overall 5-year relative survival rate is about 20% in most areas of the world, except in Japan, where 5-year survival rates of above 70% for stages I and II of gastric cancer have been reported [2]. Many studies have clearly noted that the occurrence and development of GC involve multigene dysregulation and multistep participation, which eventually leads to the evolution of cell biological behavior from normal to abnormal [3]. Therefore, it is important to identify oncogenes for the early diagnosis and treatment of cancer.

Upper gastrointestinal endoscopy is a sensitive and specific diagnostic test for gastric cancer. However, endoscopy is an interventional examination, leading to occasional compliance and poor cost-effectiveness [4]. Since peripheral blood can be noninvasively obtained and easily stored, detection of multiple serum biomarkers has become an alternative method for helping to make early diagnosis of GC. A common biomarker used for the diagnosis of GC is CEA. However, the sensitivity and specificity of CEA in GC detection are 30% and 70%, respectively. It cannot be used as a favorable diagnostic biomarker for GC. It is urgent to identify a new effective tumor marker for the diagnosis of GC.

In a previous study, we screened for GC-associated genes in GC and normal gastric tissues using a gene chip and found that SPRR2A had increased expression in GC tissues as high as 12 times (described in the supplementary data (available here)), suggesting that SPRR2A may play an important role in the development of GC [5]. The small proline-rich proteins (SPRRs) comprise a subclass of specific cornified envelope precursors encoded by a multigene family clustered within the epidermal differentiation complex region [6]. SPRR family members reported in the literature (SPRR-1, SPRR-2, or SPRR2A) exhibit elevated expression in skin and anal tumors; SPRR1 or SPRR2 expression was decreased in the skin, lung cancer, esophageal cancer, larynx cancer, and the oral cavity [7–11]. It has been reported that SPRRs are greatly regulated during wound healing and can promote cell migration [12].

In this study, we aimed to verify the overexpression of serum SPRR2A in GC with ELISA. The expression of serum SPRR2A in patients with colorectal cancer and benign gastritis was also examined. We also analyzed the sensitivity and specificity of SPRR2A as a noninvasive biomarker to improve clinical prediction of GC.

## 2. Materials and Methods

### 2.1. Patients and Sample Collection

Blood samples were collected from 390 patients admitted to the Affiliated Hangzhou First People's Hospital of Zhejiang University School of Medicine between August 2016 and May 2019. The patients included 200 with GC, 100 with gastritis, 40 with rectal cancer (RC), and 50 with colon cancer (CC). The general clinical information on all 390 subjects is shown in Table 1. Blood samples were routinely obtained in the morning within 7 to 10 days before surgery. Blood samples were also obtained from 72 patients with GC in the morning within 7 to 10 days after surgery. All samples were staged according to the 2010 American Joint Committee on Cancer (AJCC) staging system. Two pathologists confirmed the histological diagnoses and tumor-node-metastasis (TNM) stages of gastric and colorectal cancers. The various imaging, clinical laboratory, pathological examination, and clinical data of the selected subjects were recorded in detail. The inclusion criteria were as follows: (1) the patient was diagnosed as gastric cancer for the first time at the age of 33-81 years, (2) the pathological type of gastric cancer was limited to gastric adenocarcinoma, (3) no radiotherapy or chemotherapy was performed before the operation, and (4) patients were in clinical stages I-IV, with detailed clinical data and personal basic information. The exclusion criteria were as follows: (1) patients had chemotherapy or radiotherapy before surgery; (2) the patient's physical condition was poor, unable to tolerate related examinations or operations, postoperative mental condition was poor, and the prognosis was affected due to poor basic physical conditions; and (3) there were a variety of diseases, especially the second type of cancer in the body that eliminated gastric cancer.

In addition, blood samples from 100 healthy controls were collected at the Department of Medical Examination Center of the Affiliated Hangzhou First People's Hospital of Zhejiang University School of Medicine between January 2017 and May 2017. The healthy controls were confirmed to have no gastritis by gastroscopy and biopsy, and the physical examination report showed that they did not have many other diseases. We selected gastritis including chronic superficial gastritis (48 cases) and chronic atrophic gastritis (52 cases). Among them, 74 gastritis patients were HP positive and 26 were HP negative. Blood from controls was also collected in the morning. The current study was approved by the Institution Ethics Committee of the Affiliated Hangzhou First People's Hospital of Zhejiang University School of Medicine.

### 2.2. Serum Specimens

3 ml of blood was drawn from each participant in the fasting state in the morning of the first visiting day. 1 ml of serum was separated by centrifuging the blood samples at 3000*g* for 10 min, and 0.2 ml of serum was subsequently obtained after centrifugation at 10,000*g* for 5 min. The prepared serum samples were stored at -80°C.

### 2.3. ELISA Used to Quantify Serum SPRR2A

Serum SPRR2A was measured with an SPRR2A ELISA kit (X-Y Biotechnology, Shanghai, China). The standard was diluted with the original standard provided (960 pg/ml), and then the standard dilution was diluted. The standard solutions were prepared at concentrations of 30, 60, 120, 240, 480, and 960 pg/ml, and a standard curve was prepared based on the results.

After pipetting 40 *μ*l of sample buffer into each well, 10 *μ*l of the SPRR2A standard stock solution and a serum sample were added to each well. Subsequently, the plate was covered and incubated at 37°C for 30 min. A SPRR2A detection reagent was added to each well and incubated at 37°C for 30 min. The streptavidin-HRP working solution was added to each well, and then the samples were incubated at 37°C for 15 min. After washing, wells were developed with TMB, and the reaction was stopped by using a stop solution. Finally, the plates were read on a full-wave length microplate reader (MD VersaMax, Molecular Devices, CA, and USA) at 450 nm.

### 2.4. Immunoassay for CEA

Serum CEA quantitative measurements were performed at the Department of Laboratory Medicine of Hangzhou First People's Hospital of Zhejiang University School of Medicine. The concentrations of CEA in the serum samples were determined by using an Elecsys CEA kit (Abbott, IL, USA) according to the manufacturer's instructions. The tested samples were subsequently assayed on an electrochemiluminescence analyzer (ARCHITECT i2000, Abbott, IL, USA). Abnormal reference values for CEA were defined as ≥5.0 ng/ml.

### 2.5. Statistical Analysis

Comparisons of SPRR2A levels between two groups were performed by using the Mann-Whiney *U* test, and differences between the groups were compared by using the Kruskal-Wallis *H* test. The predictive ability of SPRR2A, CEA, and the combination of the 2 markers for GC was determined by logistic regression. Receiver operating characteristic (ROC) curve analysis was applied to calculate the area under the ROC curve (AUC), 95% confidence interval (CI), and Youden's index (sensitivity + specificity − 1) for each tumor marker. All the above-mentioned analyses were performed by using IBM SPSS Statistics software, version 21.0 (IBM Corp., Armonk, NY, USA). The corresponding experimental figures were drawn using GraphPad Prism 8.0 software (GraphPad Software, Inc., La Jolla, CA, USA).

## 3. Results

### 3.1. Serum SPRR2A Levels in the Five Groups of Subjects

We collected serum samples from 200 untreated patients with GC, 100 patients with gastritis, 40 patients with RC, 50 patients with CC, and 100 healthy controls. The median age of the 200 GC patients was 52 (range 33-81) years, with 126 males and 74 females, and the median absolute serum SPRR2A concentration was 124.10 (82.73-186.05) pg/ml. The median age of the 100 healthy controls was 29 (range 15~60) years, with 54 males and 46 females, and the median absolute serum SPRR2A concentration was 44.93 (28.54-74.27) pg/ml. The median age of the 100 patients with gastritis was 43 (range 23-70) years, with 58 males and 42 females, and the median absolute serum SPRR2A concentration was 50.07 (30.57-87.54) pg/ml. The median age of the patients with RC was 55 (39~72) years, with 22 males and 18 females, and the median absolute serum SPRR2A concentration was 56.58 (41.17-75.20) pg/ml. The median age of the patients with CC was 57 (31~82) years, with 39 males and 11 females, and the median absolute serum SPRR2A concentration was 62.04 (51.56-78.59) pg/ml (Table 1).

**Figure 1:**
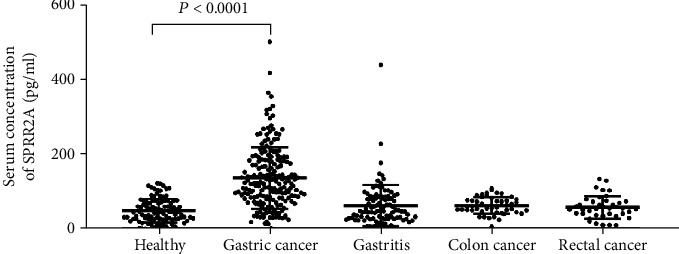
Serum SPRR2A levels in the five groups of subjects. Serum SPRR2A expression levels in the gastric cancer group (at 124.10 (82.73, 186.05)) were significantly higher than those in the gastritis group (at 50.07 (30.57, 87.54)), the colon cancer group (at 62.04 (51.56, 78.59)), the rectal cancer group (at 56.58 (41.17, 75.20)), and the healthy control group (at 44.93 (28.54, 74.27)) (*P* < 0.0001, all). There was no difference in the serum SPRR2A concentration between healthy controls and patients with gastritis, colon cancer, or rectal cancer (*P* = 0.7303, *P* = 0.5929, and *P* = 0.985, respectively).

The median serum SPRR2A concentration in all GC patients was significantly higher than those in healthy controls and gastritis and colorectal cancer patients (*P* < 0.0001, Figure 1). There was no significant difference between the healthy controls and patients with gastritis and other cancers (CC and RC, *P* > 0.05) in the serum SPRR2A concentration.

### 3.2. Relationship between Serum SPRR2A Expression Levels and the Clinicopathological Features of GC Patients

Given the significant increase in the level of serum SPRR2A for GC patients, does it relate to the process of GC development? According to the different clinicopathological factors of GC, we classified 200 GC patients. There was no significant difference between SPRR2A level and sex, age, tumor size, histological differentiation, or the Lauren classification in GC patients (*P* > 0.05) (Table 2). Significant associations between lymph node metastasis, TNM stage, and serum SPRR2A concentration were observed (*P* < 0.05). The median serum SPRR2A concentrations in patients with lymph node metastasis were significantly higher than those in patients without lymph node metastasis (*P* = 0.013). As for GC patients, SPRR2A concentration was more likely to be at an advanced stage (III stage and IV stage, *P* = 0.005).

### 3.3. Effectiveness of Serum SPRR2A Levels in Identifying GC

**Figure 2:**
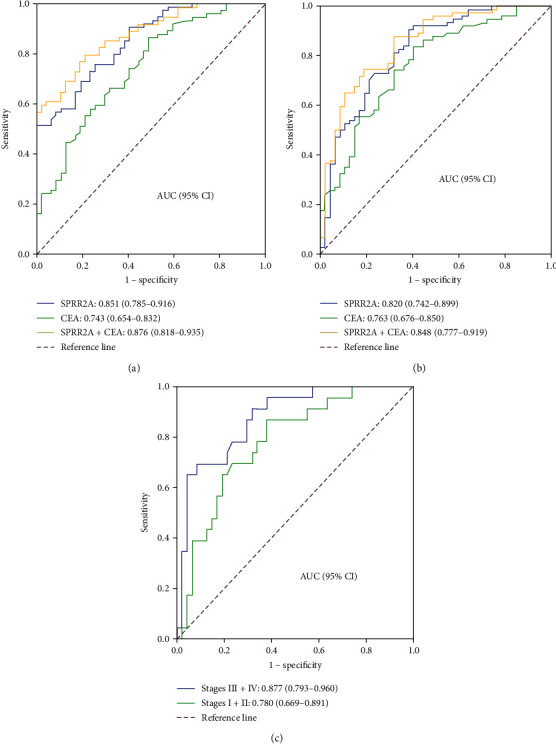
Receiver operating characteristic (ROC) curves of serum SPRR2A and CEA levels considered separately and combined for predicting gastric cancer or gastritis. (a) A serum SPRR2A concentration yielded an AUC of 0.851 (95% CI : 0.785–0.916), with 75.7% sensitivity and 74.5% specificity for discriminating GC patients from healthy people. A serum CEA concentration yielded an AUC of 0.743 (95% CI : 0.645–0.832), with 23% sensitivity and 98% specificity. The AUC for the serum SPRR2A concentration combined with the CEA concentration was 0.876 (95% CI : 0.818–0.935), with 79.7% sensitivity and 78.7% specificity. (b) Similarly, serum SPRR2A discriminated GC patients from gastritis patients with an AUC of 0.820 (95% CI : 0.742–0.899), with 90.5% sensitivity and 61.7% specificity. Serum CEA yielded an AUC of 0.763 (95% CI : 0.676–0.850), with 23% sensitivity and 97.9% specificity. The AUC for the serum SPRR2A concentration combined with the CEA concentration was 0.848 (95%CI : 0.777–0.919), with 87.8% sensitivity and 68.1% specificity. (c) The AUC for serum SPRR2A was 0.877 (95% CI: 0.793–0.960) with 87.0% sensitivity and 70.2% specificity for discriminating stage III and IV patients from healthy people. The AUC for serum SPRR2A was 0.780 (95% CI: 0.669–0.891), with 69.6% sensitivity and 68.1% specificity for stage I and II patients.

Serum SPRR2A discriminated GC patients from healthy people with an AUC of 0.851 (95%CI : 0.785–0.916). At a cut-off value of 80.7 pg/ml, the sensitivity and specificity of detection were 75.7% and 74.5%, respectively. The serum CEA concentration at the cut-off value of 5.0 ng/l yielded an AUC of 0.743 (95%CI : 0.654–0.832), with 23% sensitivity and 98% specificity. Furthermore, the AUC for the combination of SPRR2A and CEA was 0.876 (95%CI : 0.818–0.935), with 79.7% sensitivity and 78.7% specificity. Similarly, serum SPRR2A discriminated GC patients from gastritis patients with an AUC of 0.820 (95%CI : 0.742–0.899), with 90.5% sensitivity and 61.7% specificity. Serum CEA yielded an AUC of 0.763 (95%CI : 0.676–0.850), with 23% sensitivity and 97.9% specificity. The AUC for the serum SPRR2A concentration combined with the CEA concentration was 0.848 (95%CI : 0.777–0.919), with 87.8% sensitivity and 68.1% specificity (Figure 2(b)). Serum SPRR2A concentration at a cut-off value of 80.7 pg/ml yielded an AUC of 0.877 (95% CI: 0.793–0.960) with 87.0% sensitivity and 70.2% specificity for discriminating the stage III and IV patients from healthy people. In stage I and II patients, the AUC for distinguishing GC patients from healthy controls on the basis of serum SPRR2A was 0.780 (95% CI: 0.669–0.891), with 69.6% sensitivity and 68.1% specificity (Figure 2(c)).

### 3.4. Differential Serum SPRR2A Levels before and after Surgery in 72 GC Patients

**Figure 3:**
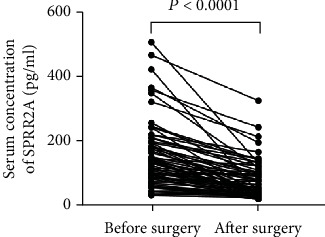
Changing trends in serum SPRR2A levels before and after surgery. Serum SPRR2A expression in gastric cancer patients postoperatively, at 38.00 (35.08, 101.05) pg/ml, was significantly lower than that in gastric cancer patients preoperatively, at 133.46 (88.04, 188.01) pg/ml (*P* < 0.001).

To determine whether SPRR2A could be used as a potential diagnostic marker for GC prior to surgery, we collected blood samples from 72 patients with GC before and after surgery, and differential SPRR2A expression was analyzed. Serum SPRR2A expression in GC patients postoperatively was 38.00 (35.08-101.05) pg/ml, which was significantly lower than that in patients preoperatively (133.46 (88.04-188.01) pg/ml) (*P* < 0.001). There was no significant difference in serum SPRR2A expression between GC patients postoperatively and healthy controls (*P* = 0.1651) (Table 3, Figure 3).

## 4. Discussion

A tumor marker was defined as a biochemical indicator that was usually found in an abnormal concentration in the presence of a tumor [13]. As discussed by Nisa et al., SPRR2A played a dual role in invasion and therapeutic resistance in head and neck squamous cell carcinoma. High expression of SPRR2A in lymph node metastases was, along with the nonoropharyngeal location of the primary tumor, an independent prognostic factor for regional disease recurrence after surgery and radiotherapy [14]. In the present study, serum SPRR2A expression in patients with GC, gastritis, and colorectal cancer and in healthy people was detected by ELISA. We found that the expression of SPRR2A was significantly increased in GC patients compared with healthy controls, while there was no significant difference between chronic gastritis and colorectal cancer patients and healthy controls. These results suggest that SPRR2A might be considered a potential tumor biomarker for GC. Then, we assessed the associations between SPRR2A expression and various clinicopathological parameters, including gender, age, tumor size, histological differentiation, Lauren classification, lymph node metastasis, and clinical stage. As a result, there was a significant association between high levels of SPRR2A and lymph node metastasis and TNM stage, but there was no significant association between SPRR2A expression and other clinicopathological characteristics.

High sensitivity is an important index in order to avoid false-negative diagnosis. Therefore, we determine the cut-off value of 80.7 pg/ml with a considerable high sensitivity of 75.7% and specificity of 74.5%. Accumulated data shows that CEA is a convenient indicator for monitoring recurrence and distant metastasis as well as for evaluating the efficacy of chemotherapy and prognosis in GC [15]. Therefore, we combined measurements of serum SPRR2A with CEA in order to improve the diagnostic efficiency for all GC patients. Marrelli et al. detected that the sensitivity in 75 recurrent cases from 133 GC patients who underwent potentially curative surgery was 44% for CEA, 56% for CA 19-9, and 51% for CA 72-4, while the combined use of CEA, CA 19-9, and CA 72-4 increased the sensitivity to 87%, which reached 100% in patients with positive preoperative levels [16]. Our study supports these recent results showing that combinations of serological biomarkers are valuable in the diagnosis of GC. We found that the combined detection of SPRR2A and CEA resulted in a significantly better AUC than those of the individual tumor markers. It is worth noting that our results showed that SPRR2A had low specificity between distinguishing gastritis and gastric cancer. Since the purpose of selecting gastritis cases was to detect whether the content of SPRR2A was different between gastric cancer and chronic gastritis, we did not select specifically chronic gastritis in our study. There were chronic superficial gastritis (48 cases) and chronic atrophic gastritis (52 cases). Many existing research results indicated that chronic atrophic gastritis was a precancerous lesion of gastric cancer; our results also showed that some patients with chronic atrophic gastritis have higher levels of SPRR2A in the blood, but due to the small number of cases, it was not certain whether SPRR2A was related to chronic atrophic gastritis. The above reasons may be the reason for the low specificity (61.7%) of SPRR2A between gastritis and gastric cancer.

Serum SPRR2A expression is significantly different in pre- and postoperative GC patients (*P* < 0.001), suggesting that serum SPRR2A may predict the prognosis of GC and aid in the postoperative monitoring of GC patients. Unfortunately, the number of specimens in gastric cancer recurrence was limited, so there is no data to show that SPRR2A is a better tumor marker than CEA in gastric cancer recurrence. In the next step, we will continue to track the prognosis of relevant patients to analyze the recurrence of SPRR2A in tumor monitoring.

While testing the diagnostic efficacy of SPRR2A in gastric cancer, we also tested its biological behavior in cells and found that SPRR2A in gastric cancer cells can promote cell proliferation and migration (data not shown). It was reported that SPRR2A was an important player in cell migration, and this migration characteristic is related to the transcriptional activity of SPRR2A regulating p53 [17]. p53 can trigger cell cycle arrest, apoptosis, and DNA repair and inhibit epithelial-mesenchymal transition (EMT). It has been reported that the upregulation of SPRR2A can inhibit p53 acetylation, prompting damaged cells to temporarily exert mesenchymal properties [18]. Although EMT plays an important role in inducing wound healing, it is also essential for the progression of cancer and metastasis. Therefore, we hypothesized that SPPR2A might promote EMT through inhibiting the acetylation of p53 during the development of GC and finally inducing infiltration and metastasis of GC cells. Further study will be needed to reveal the precise mechanism.

Although our research shows for the first time that the detection of serum SPRR2A can play a complementary role in the diagnosis of GC, especially when combined with the detection of serum CEA, there are still some limitations of this study: First, this prospective study selected a small number of gastric cancer cases and a relatively simple tissue type (only gastric adenocarcinoma). For the previous study, we found that SPRR2A mRNA in a gene chip is highly expressed in gastric adenocarcinoma. Therefore, the subjects of this study were all selected gastric adenocarcinoma patients. To further verify the role of SPRR2A in the diagnosis of gastric cancer, a retrospective study of multiple centers, large sample sizes, and multiple tissue types is also required (e.g., gastric MALT and GIST). Second, there is no strict restriction on the type of gastritis control cases, and some disease conditions that may affect serum SPRR2A levels, such as infection, ischemia, and diabetes, are not considered. In addition, although our test data shows that the serum SPRR2A level of GC patients is significantly lower than that before surgery, the change in serum SPRR2A concentration may be useful for dynamically monitoring the prognosis of GC patients undergoing surgical intervention. However, due to the small number of cases, we did not assess the prognostic value of serum SPRR2A.

## 5. Conclusion

In summary, SPRR2A showed a high diagnostic value for patients with GC; the combination of SPRR2A and CEA is a promising novel biomarker for GC diagnosis.

## Figures and Tables

**Table 1:** Serum SPRR2A and CEA levels in gastrointestinal cancer.

Characteristic	GC	Gastritis	RC	CC	Healthy
	(*n* = 200)	(*n* = 100)	(*n* = 40)	(*n* = 50)	(*n* = 100)
Age (years)	52 (33~81)	43 (23~70)	55 (39~72)	57 (31~82)	29 (15~62)
Male	126	58	22	39	54
Female	74	42	18	11	46
CEA (ng/ml)	7.66 ± 25.66	1.99 ± 1.13	24.55 ± 62.19	6.9 ± 12.42	1.58 ± 0.72
SPRR2A (ng/ml)	124.10 (82.73, 186.05)	50.07 (30.57, 87.54)	56.58 (41.17, 75.20)	62.04 (51.56, 78.59)	44.93 (28.54, 74.27)

Abbreviations: GC: gastric cancer; RC: rectal cancer; CC: colon cancer.

**Table 2:** Correlations of serum SPRR2A level and different clinicopathological parameters of GC.

Characteristic	*N* (%)	SPRR2A level	*P* value
Sex			0.192
Male	126 (63%)	129.43 (78.44, 184.08)	
Female	74 (37%)	112.87 (67.34, 160.65)	
Age (years)			0.188
<60	91 (45%)	102.52 (63.24, 145.34)	
≥60	109 (55%)	132.74 (87.63, 206.35)	
Tumor size (cm)			0.236
<5	148 (74%)	139.75 (88.33, 194.67)	
≥5	52 (26%)	111.04 (70.13, 178.35)	
Histological differentiation			0.670
Moderate	76 (38%)	110.10 (73.95, 196.41)	
Poor	124 (62%)	138.37 (88.85, 182.45)	
Lauren classification			0.227
Intestinal	69 (35%)	101.33 (60.67, 137.00)	
Diffuse	73 (36%)	143.48 (101.64, 184.65)	
Mixed	58 (29%)	113.75 (75.05, 214.55)	
Lymph node metastasis			0.013
Present	128 (64%)	164.10 (127.54, 197.00)	
Absent	72 (36%)	102.00 (65.50, 184.00)	
TNM stage			0.005
I+II	122 (61%)	113.75 (72.25, 170.04)	
III+IV	78 (39%)	180.70 (89.37, 283.30)	

The histological examination and tumor staging process were conducted by referencing the *WHO Classification of Tumours of the Digestive System*. The levels of serum SPRR2A are presented as median (IQR).

**Table 3:** SPRR2A expression levels before and after surgery in 72 GC patients.

Characteristic	SPRR2A level (pg/ml)	*P* value
Before surgery	133.46 (88.04, 188.01)	0.001
After surgery	38.00 (35.08, 101.05)	0.1651
Healthy	44.93 (28.54, 74.27)	—

## Data Availability

The datasets used and/or analyzed during the current study are available from the corresponding author on reasonable request.

## Abbreviations

SPRR2A: Serum small proline-rich protein 2A
GC: Gastric cancer
CC: Colon cancer
RC: Rectal cancer
CEA: Carcinoembryonic antigen
ROC: Receiver operating characteristic curve.

## Supplementary Materials

Supplementary Materials We screened for GC-associated genes in GC and normal gastric tissues using a gene chip and found that SPRR2A had increased expression in GC tissues as high as 12 times (described in the supplementary data), suggesting that SPRR2A may play an important role in the development of GC.
